# Structural Hypervariability of the Two Human Protein Kinase CK2 Catalytic Subunit Paralogs Revealed by Complex Structures with a Flavonol- and a Thieno[2,3-d]pyrimidine-Based Inhibitor †

**DOI:** 10.3390/ph10010009

**Published:** 2017-01-11

**Authors:** Karsten Niefind, Nils Bischoff, Andriy G. Golub, Volodymyr G. Bdzhola, Anatoliy O. Balanda, Andriy O. Prykhod’ko, Sergiy M. Yarmoluk

**Affiliations:** 1 Department für Chemie, Institut für Biochemie, Universität zu Köln, Otto-Fischer-Straße 12–14, D-50674 Köln, Germany; nils.bischoff@outlook.com; 2 Otava Ltd., 400 Applewood Crescent, Unit 100, Vaughan, ON L4K 0C3, Canada; andrew.golub@gmail.com; 3 Institute of Molecular Biology and Genetics, National Academy of Sciences of Ukraine, 150 Zabolotnogo Street, 03680 Kyiv, Ukraine; volodymyr_bdzhola@ukr.net (V.G.B.); b.anatolij@gmail.com (A.O.B.); a.o.prykhodko@gmail.com (A.O.P.); yarmolyuksm@gmail.com (S.M.Y.)

**Keywords:** protein kinase CK2, casein kinase 2, ATP-competitive inhibitors, halogen bond

## Abstract

Protein kinase CK2 is associated with a number of human diseases, among them cancer, and is therefore a target for inhibitor development in industry and academia. Six crystal structures of either CK2α, the catalytic subunit of human protein kinase CK2, or its paralog CK2α′ in complex with two ATP-competitive inhibitors—based on either a flavonol or a thieno[2,3-d]pyrimidine framework—are presented. The structures show examples for extreme structural deformations of the ATP-binding loop and its neighbourhood and of the hinge/helix αD region, i.e., of two zones of the broader ATP site environment. Thus, they supplement our picture of the conformational space available for CK2α and CK2α′. Further, they document the potential of synthetic ligands to trap unusual conformations of the enzymes and allow to envision a new generation of inhibitors that stabilize such conformations.

## 1. Introduction

“The conformational plasticity of protein kinases” [1] and its correlation with regulation were described already in 2001. The authors of this review compared eukaryotic protein kinase (EPK) structures and identified significant local structural deviations. They emphasized in that context the activation segment and the long *N*-lobal helix αC as major flexible and thus regulatory key elements. At the same time the first complex structures of EPKs with pharmacologically relevant inhibitors were published [2,3] which demonstrated that the conformational plasticity of these enzymes is even higher than imagined before. The three inhibitors described in those studies bind their target kinases (c-Abl and p38 MAP kinase) in such a way that they address a region normally occupied by the phenylalanine side chain of the “DFG” sequence motif at the *N*-terminal end of the activation segment. Later these and similar local structural states were summarized as “DFG-out” conformations [4]. In subsequent years it was more and more realized that certain EPK inhibitors do not distort their target enzymes, but rather trap them in particular conformational states that are inherent parts of complex conformational equilibria [5]. Thus, besides their pharmaceutical relevance EPK inhibitors are tools to investigate the conformational space explored by the enzymes which is larger than assumed.

In this regard protein kinase CK2—a cell-stabilizing EPK [6] accumulating in cancer cells [7] that apparently exploit CK2 activity to escape apoptosis and to assist DNA repair [8]—seemed to be an exception. Since in all known CK2 crystal structures—irrespective of whether based on the isolated catalytic subunit CK2α or on the heterotetrameric CK2α_2_β_2_ holoenzyme—the activation segment and the helix αC obtain basically the same conformation which is characteristic for an active EPK [9]. Especially, conformations equivalent to the DFG-out states of other EPKs have never been observed with CK2α and are moreover unlikely because CK2α in all of its known ortho- and paralogs contains a DWG rather than a DFG motif at the beginning of the activation segment. The central tryptophan of this motif is stabilized by many more interactions than the DFG phenylalanine of other EPKs [10].

Thus, the classical conformational switches of EPKs are not used by CK2α which is consistent to its constitutively active nature [11]. However, gradually other parts of CK2α were found to be surprisingly structurally adaptable [12,13]. Primarily for the hinge/helix αD region of human CK2α two major conformations were described [14,15] which are in a dynamic equilibrium according to metadynamics simulations [16]. In crystal structures the occurrence of these hinge/helix αD conformations is not correlated to other local conformational flexibilities [17,18]; it depends on the nature of the ATP-site ligand and on the medium used for crystallization [19]. For the glycine-rich ATP-binding loop strong or even extreme distortions from the active conformation were found [20,21,22]. And in the β3/αC loop an absolutely conserved proline was detected that is able to switch to the *cis*-peptide configuration spontaneously [23].

**Figure 1:**
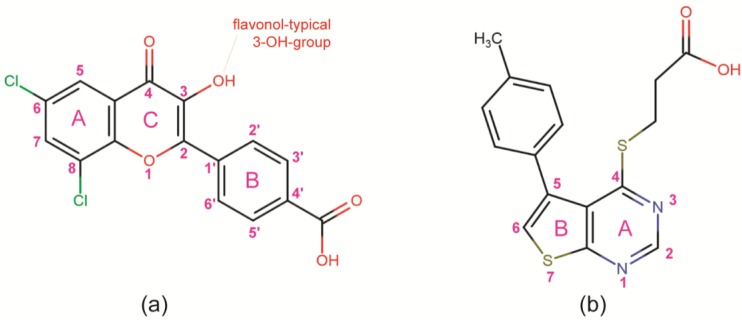
Structures of the ATP-competitive inhibitors FLC21 [28] (**a**); and TTP22 [29] (**b**) used for co-crystallization with human CK2α and/or CK2α′ constructs in this work. FLC26, the sister compound of FLC21 [22,28], which is used as a reference here (see Section 2.2), contains two bromo rather than chloro substituents at ring A attached to *C*-atoms 6 and 8.

Knowledge about the structural space the enzyme is able to explore and in particular about special local conformations in the proximity of its active site is relevant for ongoing efforts to develop highly potent and selective inhibitors of CK2 which might be beneficial to fight against hematological malignancies [24,25] as well as solid tumours [26,27]. Therefore, in this study we emphasize and extend those previous findings on local plasticities with a set of structures of both human CK2α paralogs showing partly extreme structural peculiarities. The structures were obtained by co-crystallization with two potent and selective (within a limited set of test EPKs [28,29]) ATP-competitive CK2 inhibitors: 4′-carboxy-6,8-dichloroflavonol (Figure 1a), a member of the flavonoid family of CK2 inhibitors [30] abbreviated as “FLC21” in the literature [28] and inhibiting the CK2α_2_β_2_ holoenzyme with an IC_50_ of 40 nM and a K_i_-value of 13 nM [28], and 3-{[5-(4-methylphenyl)thieno[2,3-d]pyrimidin-4-yl]thio}propanoic acid (Figure 1b)—referred to as “compound **6a**” in its original description [29] and “TTP22” in PUBCHEM (pubchem.ncbi.nlm.nih.gov/compound/1536915)—with an IC_50_ of 100 nM and a K_i_ of 40 nM [29].

## 2. Results and Discussion

### 2.1. Overview of the CK2α/CK2α′ Co-Crystal Structures

Six human CK2α/inhibitor crystal structures were determined (Table 1)—four with CK2α^1–335^, a recombinant *C*-terminally truncated version of the main paralog CK2α [31], and two with CK2α′^Asp39Gly/Cys336Ser^, a recombinant full-length construct of the isoform CK2α′ carrying an *N*-terminal (His)_6_-tag and the two point mutations Asp39Gly and Cys336Ser [32] (it should be noted that Bischoff et al. [32] erroneously failed to mention the mutation Asp39Gly, i.e., the construct “*hs*CK2α′^Cys336Ser^” referred to by Bischoff et al. [32] and tested in that work is identical with respect to the primary sequence to CK2α′^Asp39Gly/Cys336Ser^ used in this study). Notably, the two variants are unchanged in their active centre regions and in particular at the ATP cleft. Their K_M_-values for ATP were reported to be 11.2 µM in the case of CK2α^1–335^ and 11.5 µM in the case of CK2α′^Asp39Gly/Cys336Ser^ [32]. For comparison: in an extensive review Tuazon and Traugh [33] collected K_M_-values for ATP determined with 22 different non-recombinant CK2 or CK2α preparations from natural sources (mainly mammals, but in addition other vertebrates, insects, yeasts and plants); these K_M_-values range from 2 to 31 µM with an average of 13.0 µM and a standard deviation of 8.0 µM. In other words: with respect to co-substrate affinity to ATP the recombinant and mutated CK2α/CK2α′ constructs used in this study are similar to wild-type CK2 and CK2α enzymes.

The two CK2α′^Asp39Gly/Cys336Ser^ structures and two of the CK2α^1–335^ structures contain FLC21 [28] (Figure 1a) as an ATP-site ligand which allows comparisons between the two human CK2α isoforms. Two further CK2α^1–335^ structures (No. 5 and 6 in Table 1) harbour TTP22 (Figure 1b) at the co-substrate binding site. Co-crystallization experiments of TTP22 were performed with CK2α′^Asp39Gly/Cys336Ser^ as well, but they did not provide crystals of sufficient quality for X-ray diffractometry.

The monoclinic CK2α′^Asp39Gly/Cys336Ser^/FLC21 complex crystals (No. 2 in Table 1) contain two independent copies of the enzyme per asymmetric unit while the five other structures consists of only one protomer. The structures are in general of high quality; in particular the bound inhibitor molecules are clearly defined by electron density. In all cases some *N*- and *C*-terminal residues which are not relevant for ligand binding at the ATP site are flexible and not visible in the electron density maps.

### 2.2. Complex Structures with FLC21

#### 2.2.1. General Binding Mode of FLC21 to CK2α/CK2α′

**Figure 2:**
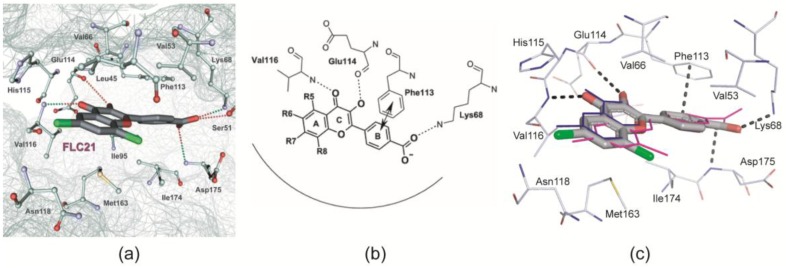
Binding mode of FLC21 to the ATP site of CK2α and CK2α′. (**a**) Section of a modelled CK2α/FLC21 complex as published by Golub et al. [28]. The picture is identical to Figure 6 in [28] © Springer Science + Business Media, LLC. 2011, and is reproduced with permission of Springer; (**b**) the four basic non-covalent interactions underlying the predicted model. The drawing is identical with Figure 1 in [28], © Springer Science+Business Media, LLC. 2011, reproduced with permission of Springer; (**c**) Section of a low-salt CK2α^1–335^/FLC21 complex (structure 3 in Table 1) drawn in an equivalent orientation and with a similar style as in Figure 2a in order to facilitate the comparison between the two pictures. The FLC21 ligands of structures 1, 2 and 4 (Table 1) were drawn with thin bonds after superimposition of the respective protein matrices.

For the binding of FLC21 to CK2α two types of advance information existed:

- Golub et al. [28] modelled FLC21 bound to CK2α as shown in Figure 2a. This CK2α/FLC21 complex model is based on a set of four predicted ionic, hydrogen bond and π/π interactions (Figure 2b). These interactions were assumed to be formed by the B and the C-ring of the flavone framework and its substituents whereas the A-ring with the two chloro atoms were supposed to be not involved.
- Guerra et al. [22] published two complex structures (PDB 4UBA and 4UB7) of CK2α^1–335^ with FLC26 [28] which is the sister compound of FLC21 containing bromo rather than chloro substituents attached to the *C*-atoms 6 and 8 (Figure 1a). These structures revealed that the inhibitor was in fact bound to the enzyme in the predicted orientation and with exactly the set of non-covalent interactions suggested in Figure 2b.

In order to characterize the binding mode of FLC21 we superimposed the five protomers of structures 1 to 4 in Table 1. As illustrated in Figure 2c the FLC21 ligands bind in identical orientations and very similar conformations irrespective of the CK2α paralog used, the crystallization condition and the crystal packing. Merely, under high-salt conditions (structure 4) the carboxy group of FLC21 and its B ring are no longer nearly coplanar (as in the other structures) but rotated against one another by about 48 degrees (magenta-coloured ligand in Figure 2c). Taken together, as observed before for FLC26 [22] the principle position and orientation of FLC21 within the ATP site and its main interactions with the enzyme are identical to the predictions of Golub et al. [28] (Figure 2a,b).

#### 2.2.2. A π-Halogen Bond Enabled by an Extremely Distorted ATP-Binding Loop

4′-Carboxyflavonol, the non-halogenated precursor compound of FLC21 and FLC26, is able to form all interactions indicated in Figure 2b and has an IC_50_ for CK2α_2_β_2_ holoenzyme inhibition of 1.3 µM [28]. The introduction of halogen substituents at positions 6 and 8 lowers this value significantly. The reported IC_50_ data are 0.18 µM and 0.08 µM for the mono-halogenated compounds 6-chloro-4’-carboxyflavonol and 6-bromo-4′-carboxyflavonol as well as 0.04 µM and 0.008 µM for the di-halogenated inhibitors FLC21 and FLC26 [28]. In other words: to substitute the 6- and the 8-position with halogen atoms increases the inhibitory power although these halogen substituents point away from the hinge/helix αD region and are thus—unlike those of many typical halogenated EPK inhibitors [35]—unable to form halogen bonds with the peptide backbone (Figure 2a,b); a further conclusion from the inhibition data is that introducing bromine is more effective than chlorine.

**Figure 3:**
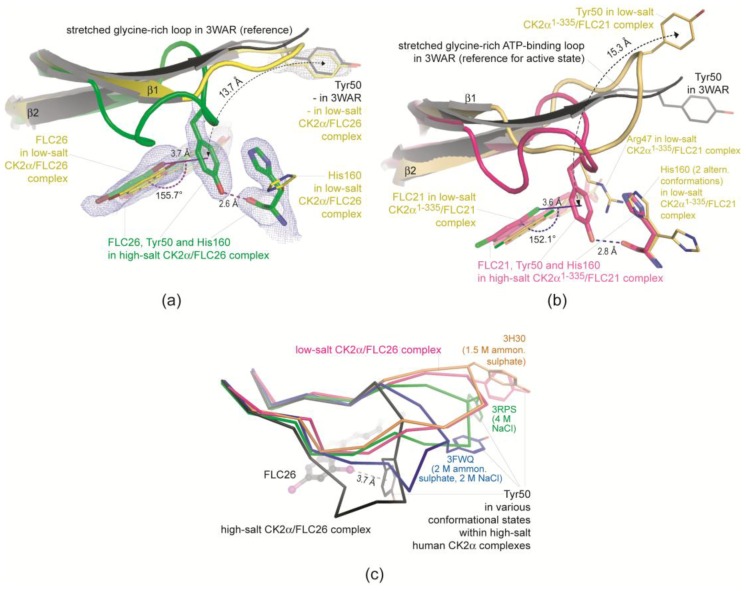
Formation of a kosmotropic-salt supported π-halogen bond between either FLC21 or FLC26 and CK2α^1–335^. (**a**) FLC26: Under high-salt crystallization conditions Tyr50 at the tip of the ATP-binding loop bends down to the Br8 atom of FLC26 (π-halogen bond) and His160 (hydrogen bond); (**b**) In the case of FLC21 the same phenomenon is found under high-salt conditions (structure 4 of Table 1; parts with magenta-coloured *C*-atoms). Under low-salt conditions (structure 3 of Table 1; parts with yellow *C*-atoms) Arg47 replaces Tyr50 in the space between FLC21 and His160 which was not observed for FLC26; (**c**) ATP-binding loops in human CK2α structures obtained from various high-salt crystallization conditions. The strong distortion of the ATP-binding loop observed in the complexes with FLC26 and FLC21 is not exclusively caused by the high salt concentration since it was never found in any high-salt structure of CK2α published previously. Parts (**a**) and (**c**) of the figure are reprinted with kind permission from Guerra et al. [22]. Copyright (2015) American Chemical Society.

For FLC26 these observations were rationalized with the two aforementioned complex structures 4UB7 and 4UBA [22]. One of these structures—obtained from high-salt crystallization conditions—showed a π-halogen bond between the Br8-atom and the aromatic ring of the Tyr50 which was only possible after a dramatic conformational change of the ATP-binding loop (Figure 3a). With low-salt crystallization conditions this π-halogen bond was absent in the crystalline state but kinetic studies [22] suggested that it nevertheless contributes to the inhibitory efficacy of FLC26 in the solute state.

One aim of the study presented here was to test if FLC21 with its chloro instead of bromo substituents can form this remarkable π-halogen bond as well. An answer to this question is given in Figure 3: the high-salt CK2α^1–335^/FLC21 structure (magenta-coloured structure in Figure 3b; No. 4 in Table 1) is very similar to the CK2α^1–335^/FLC26 structure (green structure in Figure 3a) and contains in particular—and in contrast to its low-salt pendant (No. 3 in Table 1)—the π-halogen bond with Tyr50 in question. This π-halogen bond requires an extreme distortion of the ATP-binding loop (Figure 3c) which is found—with exception of the high-salt CK2α^1–335^/FLC26 structure (Figure 3a)—in no other previously reported CK2α structure, in particular in none of the known high-salt structures (Figure 3c). Obviously, neither a properly located halogen substituent nor a high concentration of a kosmotropic salt alone is sufficient to establish this particular arrangement. Rather, both conditions must be matched simultaneously to capture this feature.

#### 2.2.3. FLC21 Traps the Gly-Rich Loop Arginine of CK2α and CK2α′ in a Non-Functional Conformation

**Figure 4:**
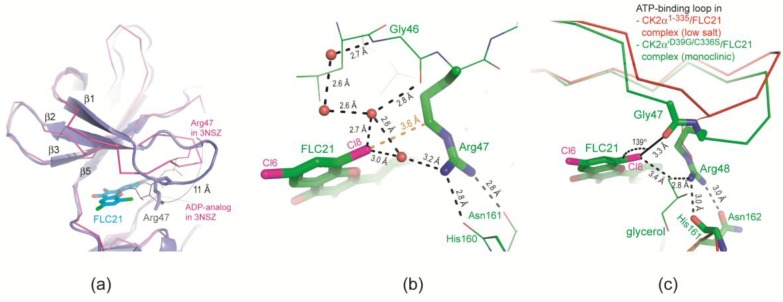
Arg47/48 and FLC21 stabilize a non-functional conformation of the ATP-binding loop. (**a**) The *N*-terminal domain of the low-salt CK2α^1–335^/FLC21 complex (purple; structure 3 in Table 1) and for comparison (in order to illustrate the functional state of the ATP-binding loop) of PDB-file 3NSZ which contains human CK2α^2–331^ in complex with an ADP-analog [37]; (**b**) More detailed and focused view of the low-salt CK2α^1–335^/FLC21 complex in which the hydrogen-bonds of Gly46, Arg47 and the Cl8-atom of FLC21 are highlighted. The nearest atomic distance between FLC21 and Arg47 is indicated in orange colour. Four water molecules are drawn as red balls; (**c**) the equivalent region in protomer B of the monoclinic CK2α′^Asp39Gly/Cys336Ser^/FLC21 complex structure (No. 2 in Table 1). For comparison the ATP-binding loop plus Arg47 side chain of the low-salt CK2α^1–335^/FLC21 complex (structure 3 in Table 1) is drawn in red colour. Note that due to a one residue insertion near the *N*-terminus the sequence numbers in human CK2α′ are shifted by +1 compared to human CK2α.

While the high-salt CK2α^1–335^ structures of FLC21 and FLC26 resemble each other right up to atomic details, there is a conspicuous local difference between the two low-salt structures: in the low-salt CK2α^1–335^/FLC26 structure (yellow structure in Figure 3a) the glycine-rich ATP-binding loop adopts a stretched conformation resembling the atomic resolution CK2α apo structure 3WAR [36] (black structure in Figure 3a); in contrast in complex with FLC21 Arg47, a loop member flanked by two glycine residues and thus conferred with a significant adaptability, is bent down towards the *C*-terminal domain (Figure 3b and Figure 4a) where it forms hydrogen bonds to His160 and Asn161 (Figure 4b).

This is a non-functional state of the ATP-binding loop because the Arg47 side chain interferes with the ribose region of the canonical ATP site (Figure 4a). FLC21 seems to support this local conformation since it is present in the two FLC21 complex structures with CK2α′^Asp39Gly/Cys336Ser^ (No. 1 and 2 in Table 1) as well albeit with more internal flexibility, i.e., less well defined electron densities. So far this “Arg47-down” conformation did not occur in complexes with FLC26; if this reflects a significant difference between FLC21 and FLC26 (which cannot be clarified currently due to the limited number of complex structures with FLC26), this would be surprising because it is the Cl8 atom of FLC21 that is in atomic contact to the Arg47 side chain (Arg48 in CK2α′) and because bromine—the Cl8 equivalent in FLC26—generally forms stronger halogen bonds than chlorine [38]. 

In fact, a closer inspection of the two best defined cases of the FLC21-Arg47/48 arrangement shows that halogen bonding plays a role as a stabilizing factor but that it is not the only one:

- In the low-salt CK2α^1–335^/FLC21 structure a network of hydrogen bonds around the Cl8 atom stabilizes the conformation; the propensity to operate as hydrogen bond acceptor is for chloro in fact higher than for bromo substituents (Figure 4b).
- Figure 4c was drawn from one of the two protomers of the monoclinic CK2α′^Asp39Gly/Cys336Ser^ complex structure with FLC21. Here, the Arg48 side chain is hydrogen-bonded to His161 and Asn162 (the CK2α′ equivalents of His160 and Asn161 in CK2α) similar to what is seen in Figure 4b. However, in addition the Cl8 atom forms a geometrically well-established halogen bond with a peptide oxygen of the ATP-binding loop, namely the peptide group connecting Gly47 and Arg48 (Figure 4c). There is no reason to believe that FLC26 binding cannot support such a particular state via a halogen bond as well; co-crystallization efforts of FLC26 with CK2α′^Asp39Gly/Cys336Ser^ might clarify this.

A similar halogen bond, namely between an inhibitor and the backbone of the ATP-binding loop, was not observed in any CK2α/CK2α′ structure before.

#### 2.2.4. Prolyl *cis/trans*-Isomerization at the β3/αC Loop

**Figure 5:**
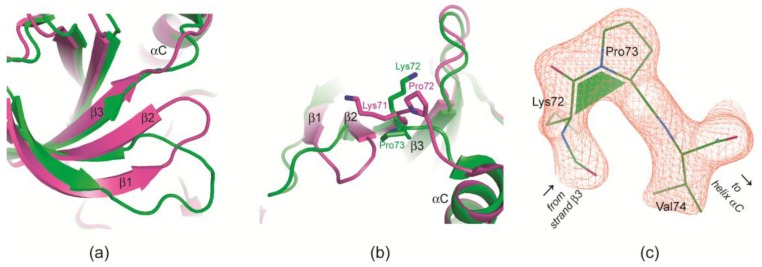
Strong deformations of the ATP-binding loop of CK2α/CK2α′ can be correlated with *cis*-peptide formation at the central proline residue of the β3/αC loop. (**a**,**b**) Overlay of protomer B of the monoclinic CK2α′^Asp39Gly/Cys336Ser^/FLC21 complex structure (structure 2 in Table 1; green) and a high-resolution structure of human CK2α^2–331^ in complex with an ADP-analogue (PDB 3NSZ [37]; magenta). The canonical β-sheet of the *N*-terminal domain is drawn in two different orientations; (**c**) the β3/αC loop in protomer B of the monoclinic CK2α′^Asp39Gly/Cys336Ser^/FLC21 complex structure covered by electron density (cutoff level 1.5 σ).

Just like the π-halogen bonds with FLC26 and FLC21 visible in Figure 3a,b, respectively, the unusual halogen bond illustrated in Figure 4c requires a strong deformation of the ATP-binding loop which is only possible if the β2-strand is partly resolved from the next strand (β3) of the canonical *N*-lobal β-sheet. This reduction of strands β1 and β2 and the inclination of the interconnecting ATP-binding loop away from the canonical β-sheet are illustrated in Figure 5a.

In this context the *C*-terminal part of the neighbouring strand β3 is particularly critical. Here, the final residue Lys72 (Lys71 in CK2α) normally stabilizes the functional conformation of the ATP-binding loop first by a β-sheet-typical main chain/main chain hydrogen bond and second by stretching its side chain over the strand β2 as visible in Figure 5b. Without these contacts Lys71/72 is free for structural alternatives. It can even force its extended side chain into a completely different direction (Figure 5b) which is accomplished by a *cis*-peptide bond with the succeeding proline residue, the central residue of the β3/αC loop (Figure 5c). A cis-peptide bond at the equivalent position was observed only once before, namely in a CK2α^1–335^ structure with a cyclic peptide bound to the CK2β interface [23]. Here, it occurs—well documented by electron density (Figure 5c)—in protomer B of the CK2α′^Asp39Gly/Cys336Ser^/FLC21 complex structure, i.e., for the first time with CK2α′ and remarkably enough in correlation with a novel halogen bond (Figure 4c) and with the strongest ATP-binding loop deviation in any CK2α/CK2α′ structure obtained so far under low-salt crystallization conditions.

In summary, the two halogenated flavonol compounds FLC21 and FLC26 [28] dispose of a remarkable potential to trap and stabilize CK2α/CK2α′ in extraordinary conformations characterized by extreme deviations of the ATP-binding loop and by unusual halogen bonds between this loop and the inhibitor. So far, this was already known for the bromo compound FLC26 [22], but only for a single structure of human CK2α obtained from largely artificial high-salt crystallization conditions. The results reported here confirm this impression; more important, they extend it to the chloro compound FLC21 on the inhibitor side, to the paralog CK2α′ on the protein side, to the structural environment of the ATP-binding loop (β3/αC-loop) and finally to low-salt crystallization conditions that are significantly closer to the physiological milieu.

#### 2.2.5. The Hinge/Helix αD Region Harbours a Novel αD Site

**Figure 6:**
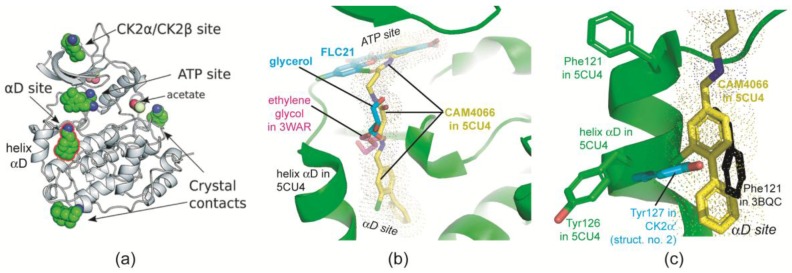
Molecular fragments at a novel αD site, at the ATP site and along the interconnecting path. (**a**) Brear et al. [39] found 2-(3,4-dichlorophenyl)ethanamine (green balls for carbon atoms) at several cavities of CK2α and identified in this way the αD site. The picture was taken and slightly modified from [39], published by The Royal Society of Chemistry; (**b**) Section of a complex structure of CK2α with the bivalent inhibitor CAM4066 (PDB 5CU4 [39], CAM4066 with yellow carbon atoms). After structural superimposition elements of PDB file 3WAR [36] (ethylene glycol, magenta coloured *C*-atoms) and of chain B of the monoclinic CK2α′^Asp39Gly/Cys336Ser^/FLC21 complex (light blue *C*-atoms) are drawn; (**c**) The αD site is occupied by Phe121 in CK2α structures with closed hinge/helix αD conformation (here represented by 3BQC; black) or partially by Tyr126 (Tyr127 in CK2α′; here drawn in light blue from structure 2 of Table 1) in structures with open hinge/helix αD conformation. αD site ligands like CAM4066 replace both of them.

A second remarkable detail of Figure 4c—apart from the novel FLC21/Gly47 halogen bond—is a glycerol molecule that acquires the role of a water molecule to form a hydrogen bond bridge between FLC21 and the side chain of Arg48. At a first glance this is not more than an artefact from the cryo solution used to prepare the monoclinic CK2α′^Asp39Gly/Cys336Ser^/FLC21 complex crystals for X-ray diffractometry. However, this observation gains stronger significance in the light of Figure 6a that is overtaken from a recent study by Brear et al. [39]. These authors crystallized a human CK2α construct (with an *N*-terminal extension which is not relevant in this context) together with the small benzene derivative 2-(3,4-dichlorophenyl)ethanamine. They found this fragment attached to several sites (Figure 6a), among them crystal contacts but also inherent binding sites of the enzyme.

Most interesting is a novel “αD site” behind the small helix αD (Figure 6a) and with access to the ATP site. Brear et al. [39] noticed that ethylene glycol can occupy the entrance region of the αD site (found in PDB 3WAR [36]) and exploited all findings about molecular fragments to design the bivalent CK2 inhibitor CAM4066. CAM4066 occupies the ATP site and the αD site simultaneously (Figure 6b). An overlay of a CK2α/CAM4066 co-crystal structure (PDB 5CU4) with chain B of the monoclinic CK2α′^Asp39Gly/Cys336Ser^/FLC21 reveals that the aforementioned glycerol molecule is attached to the connecting path between the two sites (Figure 6b). Thus, it supplements the set of molecular fragments that can be combined to similar bivalent inhibitors.

The αD site is a consequence of a structural flexibility of the hinge/helix αD region that was noticed some years ago [9,12,15]. Two main local conformations were identified and interpreted in the light of the spine concept for EPK regulation [18,40], the less frequent “closed” conformation in which Phe121 is an integral part of a stack of hydrophobic residues called “catalytic spine” [41] and the predominant “open” conformation with Tyr126 (Tyr127 in CK2α′) occupying at least partially the spine region (Figure 6c). Based on these crystallographic findings Klopffleisch et al. [19] anticipated that there is “a dynamic conformational equilibrium in solution that can be resolved by suitable ligands.” Brear et al. [39] were the first now to identify such ligands and to demonstrate their potential for selective inhibition of CK2. In the sense of spine concept one can say: these compounds replace either Phe121 or Tyr126 from their catalytic spine locations, complete the catalytic spine themselves and create thus the αD site.

### 2.3. Complex Structures with TTP22

#### 2.3.1. CK2α Binds TTP22 as Predicted under Low-Salt, but Differently under High-Salt Conditions

**Figure 7:**
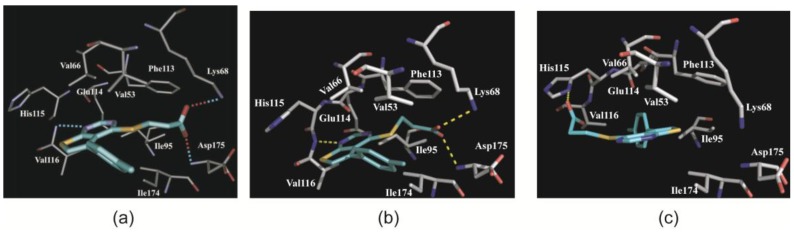
Comparison of modelled and experimental complex structures of TTP22 with CK2α. (**a**) In-silico model of a CK2α/TTP22 complex [29]. The picture was reproduced from Golub et al. [29] with kind permission by Elsevier B.V; (**b**) The same section and orientation as in (**a**), but now obtained from the experimental low-salt CK2α^1–335^/TTP22 complex structure (No. 5 of Table 1); (**c**) The same section and orientation as in (**a**) and (**b**), but now obtained from the experimental high-salt CK2α^1–335^/TTP22 complex structure (No. 6 of Table 1). Pictures (**b**) and (**c**) were designed in a similar style as panel (**a**) in order to enable easy comparisons.

Like FLC21, TTP22 (Figure 1b) was theoretically docked to CK2α in the original description of the compound [29]. The carboxy group of the TTP22 molecule was assumed to form hydrogen bonds to Lys68 and to Asp175 (Figure 7a) in a similar way as FLC21 (Figure 2a). A further predicted anchor point was the N1-atom within the pyrimidine ring A which was supposed to be hydrogen bonded to the hinge backbone (Val116). The thienopropionic acid substituent has an optimal length to allow all three hydrogen bonds simultaneously [29]. In this orientation the aromatic substituent of the B-ring points to the solvent but is nevertheless flanked by some hydrophobic side chains as visible in Figure 7a.

**Figure 8:**
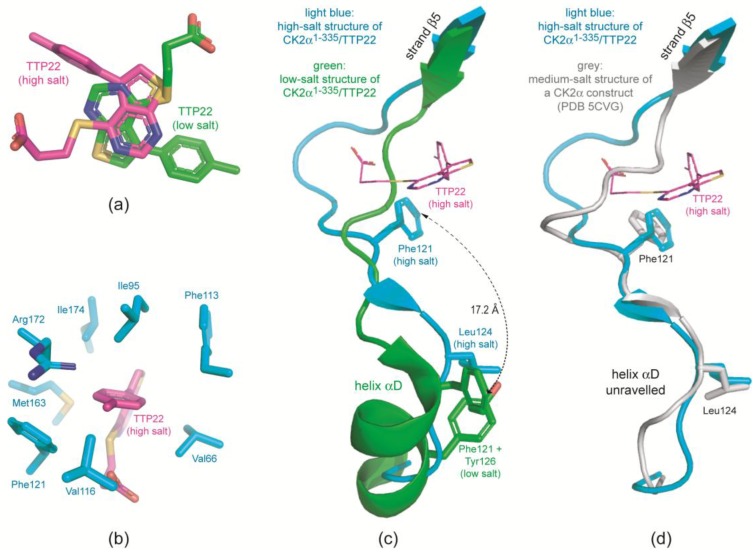
Structural differences between the high-salt and the low-salt CK2α^1–335^/TTP22 complex. (**a**) The inhibitor TTP22 bound to CK2α^1–335^ under low-salt conditions (structure 5 of Table 1; magenta-coloured *C*-atoms) and under high-salt conditions (structure 6 of Table 1; green *C*-atoms) after superimposition of the protein matrices; (**b**) Cage of hydrophobic side chains or side chains with an aliphatic part (Arg172) surrounding the methylphenyl moiety of TTP22 under high-salt conditions; (**c**) Comparison of the hinge/helix αD region of the low-salt (green) and the high-salt (light blue) CK2α^1–335^/TTP22 structure; (**d**) Comparison of the hinge/helix αD region of the high-salt CK2α^1–335^/TTP22 structure (light blue) and a recently published medium-salt CK2α structure with unraveled helix αD (PDB 5CVG [39]).

A look at the low-salt CK2α^1–335^/TTP22 structure (No. 5 of Table 1) revealed that this prediction including all three hydrogen bonds was essentially correct. To illustrate this fact we designed a figure (Figure 7b) with the same orientation, structural elements and background as in Figure 7a. However, in contrast to FLC21 the binding mode of TTP22 changes dramatically under high-salt conditions (Figure 7c). Compared to the low-salt state the inhibitor turned by about 180 degrees around an axis perpendicular to the plane formed by rings A and B (Figure 8a). In this way the carboxy moiety is no longer in the proximity of Lys68, but now of the interdomain hinge member His115 (Figure 7c). Concerning the charges this makes sense because at the pH-value of the crystallization medium (about 5.0) the His115 side chain is protonated and positively charged while the carboxy group of TTP22 is still deprotonated. Vice versa, the methylphenyl substituent of ring B changes its direction from outside to inside. It is now (i.e., under high-salt conditions) perfectly embedded in a hydrophobic environment (Figure 8b). This observation fits nicely to the well-known fact that hydrophobic interactions are supported by kosmotropic salts that interact strongly with water and disturb thus the ordered water shell around hydrophobic patches in an aqueous environment.

#### 2.3.2. TTP22 Traps CK2α with an Unraveled Helix αD under High-Salt Conditions

A conspicuous feature in Figure 8b is the fact that Phe121 is part of the hydrophobic cage around the methylphenyl substituent of TTP22. This is quite surprising because it requires a movement of Phe121 by more than 17 Å compared to the low-salt CK2α^1–335^/TTP22 complex (Figure 8c). In this low-salt structure Phe121 has its typical and well known position of an open hinge/helix αD conformation while Tyr126 occupies—also quite usual—the catalytic spine cavity (i.e., the αD site according to Brear et al. [39]). According to all experiences obtained so far (see in particular the analysis of Klopffleisch et al. [19]) a change to a high-salt medium should induce a switch to the closed hinge/helix αD conformation in which Phe121 completes the catalytic spine and harbours its side chain in the αD site (see black side chain in Figure 6c).

In the high-salt CK2α^1–335^/TTP22 structure, however, something different happens: Phe121 moves as indicated in Figure 8c and gets in contact to the methylphenyl moiety of the inhibitor. Simultaneously Leu124 occupies the catalytic spine position (αD site) (Figure 8c). These drastic changes are only possible if the helix αD is completely unraveled as visible in Figure 8c. When we observed this instance of “hypervariability” of the hinge/helix αD region we first considered it as an interesting but completely artificial feature induced by the combination by the high salt concentration (>4 M NaCl) and a suitable ligand. However, in the recent publication by Brear et al. [39] a similar conformation with Phe121 and Leu124 at equivalent positions was reported (Figure 8d). This structure (PDB 5CVG) was obtained from crystals grown with a non-salt precipitant albeit with a significant addition (0.75 M) of ammonium acetate. Insofar it is possible, that such extreme conformations of the hinge/helix αD region belong to the normal conformational space explored by CK2α and that it just requires suitable ligands and crystallization conditions to trap them.

The hinge/helix αD region of CK2α was a discussion point already in the very first CK2 structure publication [42]. Since that time it has caused surprises repeatedly, and it seems as if it continues to do so. In particular, recent developments allow the vision that the unique nature of this region compared to other EPKs can be exploited to design CK2 inhibitors of high selectivity.

## 3. Materials and Methods

### 3.1. CK2 Inhibitors

The CK2 inhibitors FLC21 and TTP22 were synthesized as described previously [28,29].

### 3.2. Proteins

The two enzyme constructs CK2α^1–335^ and CK2α′^Asp39Gly/Cys336Ser^ were prepared as described previously [15,32]. In the case of CK2α′^Asp39Gly/Cys336Ser^ it should be noted that the point mutation Cys336Ser was planed to improve the protein solubility while the Asp39Gly mutation occurred unintendedly during PCR and was overlooked in spite of sequencing. Nevertheless the construct CK2α′^Asp39Gly/Cys336Ser^ was used here because it showed significant catalytic activity with a K_M_-value for ATP of 11.5 µM [32] which is comparable to wild-type CK2 and CK2α preparations [33] and because the mutated position is remote from the ATP site and its environment (see more detailled discussion in Section 2.1). The proteins were stored in stock solutions containing 500 mM NaCl, 25 mM Tris/HCl, pH 8.5, as a background. The final protein mass concentrations (determined via UV-absorption at 280 nm) were 6.0 mg/mL for CK2α^1–335^ and 5.5 mg/mL for CK2α′^Asp39Gly/Cys336Ser^.

### 3.3. Crystallization

As indicated in Table 1 the crystallization efforts began in either case with mixing 90 µL protein solution with 10 µL 10 mM inhibitor (FLC21 or TTP22) solution in DMSO, incubating this mixture for at least 30 min at room temperature or—in the case of structure 3 of Table 1—on ice. After incubation precipitated material was removed by centrifucation. For crystallization according to the sitting drop variant of the vapour diffusion method 1 µL pre-incubated protein/inhibitor solution was mixed with 1 µL, respectively, of various reservoir solutions. All CK2α^1–335^ crystallization drops equilibrated at 20 °C and all CK2α′^Asp39Gly/Cys336Ser^ crystallization drops at 4 °C. Optimal crystal growth was observed with the reservoir compositions given in Table 1.

### 3.4. X-Ray Diffractometry

The CK2α^1–335^/inhibitor and CK2α′^Asp39Gly/Cys336Ser^/inhibitor crystals were flash frozen in liquid nitrogen and mounted for X-ray diffractometry at 100 K. The CK2α^1–335^ crystals grown under high-salt conditions (structures 4 and 6 of Table 1) were directly transferred to liquid nitrogen without a special cryo colution. In the other four cases cryo solutions were prepared which were basically the reservoir solution, respectively, enriched with glycerol and through which the crystals were shortly drawn prior to the transfer to liquid nitrogen. The final glycerol concentrations of the cryo solutions were 20% (*v*/*v*) for structure 1 of Table 1, 25% (*v*/*v*) for structure 2 and 15% (*v*/*v*) for structures 3 and 5.

X-ray diffraction data were measured with a microfocus rotating copper anode X-ray diffractometer (MicroMax-007 from Rigaku, Tokyo, Japan) and with three synchrotron beamlines (beamline ID23-1 at the ESRF in Grenoble, France, beamline X06DA at the Swiss Light Source, Paul Scherrer Institut, in Villigen, Switzerland, and beamline MX-14.1 of HZB BESSY II, Helmholtz-Zentrum Berlin, Germany). Final diffraction data sets were collected as indicated in Table 1. All diffraction data sets were processed with XDS [43] for indexing and integration and with AIMLESS and CTRUNCATE of the CCP4 software package [44] for scaling and conversion to structure factor amplitudes.

### 3.5. Structure Solution, Refinement, Validation and Deposition

The structures were solved by molecular replacement with PHASER [45] and refined and validated with PHENIX [46]. The inhibitor ligands FLC21 and TTP22 were parameterised with PRODRG [47]. Manual corrections were performed with COOT [48]. The final structures were deposited at the Protein Data Bank and are available under the accession codes indicated in Table 1.

### 3.6. Illustration

Figure 5c was drawn with COOT [48]. All other illustrations—if not taken from the sources indicated in the respective figure legends—were prepared with PYMOL [49].

## 4. Conclusions

Previous analyses [9] have shown that CK2α—in spite of a conspicuous overall rigidity in comparison to other EPKs—has structurally rather variable regions. One of them—the ATP-binding loop—is well-known among EPKs for its conformational adaptability, another—the hinge/helix αD region—is in this respect unique for CK2α. The results presented here intensify this picture. They document structural snapshots reflecting, confirming and partially explaining a hypervariability in these two zones that only emerged in recent years. These findings are undoubtedly relevant for the development of CK2 inhibitors: they offer new strategies to improve selectivity by the combination of binding sites as already shown [39] or by stabilizing the enzyme in unique non-functional states and thus pave the way to a new generation of CK2 inhibitors.

## Figures and Tables

**Table 1:** Crystallization, X-ray diffraction data and refinement statistics.

Structure No.		1	2	3	4	5	6
PDB Code		5M4U	5M56	5M4F	5M4I	5M4C	5M44
**Crystallization**							
Crystallized complex		CK2α′^Asp39Gly/Cys336Ser^ + FLC21		CK2α^1–335^ + FLC21		CK2α^1–335^ + TTP22	
Vapour diffusion reservoir composition		25% PEG5000 MME, 0.2 M ammonium sulphate, 0.1 M MES, pH 6.5	25% PEG4000, 15% glycerol, 0.17 M sodium acetate, 0.08 M Tris/HCl, pH 8.5	24% PEG3350, 0.2 M KCl	4.3 M NaCl, 0.1 M sodium citrate, pH 5.2	24% PEG8000, 0.2 M KCl	4.2 M NaCl, 0.1 M sodium citrate, pH 5.0
Sitting drop composition before equilibration		1 µL reservoir + 1 µL enzyme/FLC21 mixture (90 µL 5.5 mg/mL enzyme, 0.5 M NaCl, 25 mM Tris/HCl, pH 8.5, mixed and pre-equilibrated with 10 µL 10 mM FLC21 in DMSO)		1 µL reservoir + 1 µL enzyme/inhibitor mixture (90 µL 6 mg/mL enzyme, 0.5 M NaCl, 25 mM Tris/HCl, pH 8.5, mixed and pre-equilibrated with 10 µL 10 mM inhibitor in DMSO)			
**X-ray Diffraction Data Collection**							
Wavelength (Å)		1.0000	1.0000	0.91841	0.91841	1.0000	1.54179
Synchrotron (beamline)		SLS (X06DA)	SLS (X06DA)	HZB BESSY II (MX-14.1 [34])	HZB BESSY II (MX-14.1 [34])	SLS (X06DA)	Home source (rot. Cu anode)
Space group		P2_1_2_1_2_1_	P2_1_	P2_1_2_1_2_1_	P4_3_2_1_2	P2_1_2_1_2_1_	P4_3_2_1_2
Unit cell	a, b, c (Å)	46.85, 83.78, 142.34	69.34, 87.62, 72.98	48.03, 79.57, 82.14	72.59, 72.59, 133.25	48.10, 79.42, 82.34	72.06, 72.06, 131.58
	α, β, γ (°)	90.0, 90.0, 90.0	90, 109.69, 90	90.0, 90.0, 90.0	90.0, 90.0, 90.0	90.0, 90.0, 90.0	90.0, 90.0, 90.0
Protomers per asymmetric unit		1	2	1	1	1	1
Resolution (Å) (highest res. shell)		44.50–2.195 (2.274–2.195) ^1^	40.94–2.237 (2.317–2.237)^ 1^	41.12–1.519 (1.574–1.519)^ 1^	37.89–2.218 (2.297–2.218)^ 1^	41.14–1.935 (2.004–1.935)^ 1^	27.84–2.710 (2.807–2.710)^ 1^
R_sym_ (%)		19.1 (118.5)^ 1^	9.3 (65.7)^ 1^	5.9 (78.7)^ 1^	11.1 (116.9)^ 1^	9.8 (73.1)^ 1^	13.1 (80.8)^ 1^
CC1/2		0.993 (0.684)^ 1^	0.996 (0.685)^ 1^	0.999 (0.661)^ 1^	0.999 (0.616)^ 1^	0.998 (0.758)^ 1^	0.996 (0.693)^ 1^
Signal-to-noise ratio (I/σ_I_)		9.99 (1.72)^ 1^	9.76 (1.78)^ 1^	15.82 (1.89)^ 1^	15.35 (1.84)^ 1^	15.25 (2.26)^ 1^	15.75 (2.32)^ 1^
No. of unique refl.		29246 (2680)^ 1^	39,108 (3544)^ 1^	49,151 (4808)^ 1^	18,350 (1795)^ 1^	23,280 (1476)^ 1^	9935 (947)^ 1^
Completeness (%)		99.0 (93.0)^ 1^	98.0 (90.0)^ 1^	100.0 (99.0)^ 1^	100.0 (100.0)^ 1^	96.0 (62.0)^ 1^	100.0 (98.0)^ 1^
Multiplicity		6.4 (5.6)^ 1^	3.3 (2.9)^ 1^	4.1 (4.0)^ 1^	7.9 (7.9)^ 1^	6.3 (5.2)^ 1^	6.9 (5.9)^ 1^
Wilson B-fact (Å^2^)		21.44	29.76	15.41	36.83	21.84	40.32
**Structure Refinement and Validation**							
No. of reflections for R_work_/R_free_		1142	37,938/1161	48,116/1031	17,314/1034	22,177/1104	8979/956
R_work_/R_free_ (%)		21.41/17.04	16.35/20.64	16.28/18.26	18.80/22.83	15.64/19.68	21.86/25.94
Number of non-H-atoms		3175	5987	3248	2936	3069	2820
Protein		2789	5537	2821	2798	2806	2782
Ligand/ion		71	72	43	28	35	27
Water		315	378	384	110	228	11
Aver. B-factor (Å^2^)		28.61	38.76	20.89	52.22	28.55	52.32
Protein		27.33	38.57	19.30	52.64	28.01	52.52
Ligand/ion		43.49	40.20	26.19	45.09	27.14	44.05
water		36.62	41.20	31.95	43.45	35.37	22.12
RMS deviations							
Bond lengths (Å)		0.003	0.002	0.014	0.002	0.009	0.002
Bond angles (°)		0.570	0.50	1.28	0.46	0.96	0.45
Ramachandran plot							
favoured (%)		97.0	95.9	97.9	96.4	97.6	95.4
allowed (%)		2.7	3.8	1.8	3.6	2.1	4.0
outliers (%)		0.3	0.3	0.3	0.0	0.3	0.6

^1^ Values in brackets refer to the highest resolution shell.
